# Biosurveillance and early outbreak detection of rabies in settings with limited laboratory capacity using spatiotemporal clustering and a machine learning framework

**DOI:** 10.1038/s41598-026-55346-7

**Published:** 2026-05-28

**Authors:** Ravikiran Keshavamurthy, Jesse Blanton, Lillian Orciari, Andrew D. Gibson, Pierre Dilius, Haim Joseph, Ryan M. Wallace

**Affiliations:** 1 Poxvirus and Rabies Branch, Division of High Consequence Pathogens and Pathology, National Center for Emerging and Zoonotic Infectious Diseases, Centers for Disease Control and Prevention, 1600 Clifton Rd NE, Atlanta, GA 30329-4027 USA; 2 Rural Development and Natural Resources, Haiti Ministry of Agriculture, Port au Prince, Haiti; 3 Mission Rabies, Cranborne, Dorset, UK

**Keywords:** Rabies, Zoonoses, Machine learning, Disease surveillance, Extreme gradient boosting, Spatiotemporal clustering, Situational awareness, Diseases, Ecology, Ecology, Health care, Microbiology

## Abstract

Rabies is an important but often neglected zoonotic disease with significant public health, veterinary, and economic impacts. Inadequate surveillance and diagnostic resources in low- and middle-income countries impede its effective public health responses. This study demonstrates the use of an extreme gradient boosting (XGB) technique for animal rabies risk assessment in endemic areas of Haiti with limited diagnostic resources. We employed spatiotemporal clustering and trend analysis techniques to assess rabies status across large geographic areas. The XGB model achieved high specificity (0.99, 95% CI: 0.98–0.99), sensitivity (0.78, 95% CI: 0.61–0.95), negative predictive value (1.0, 95% CI: 0.99–1.00), and accuracy (0.98, 95% CI: 0.98–0.99) in predicting animal rabies cases. The framework identified 20 high-risk rabies clusters, representing a 40% increase in detected transmission zones compared to laboratory-confirmed data alone. This included 8 clusters in underserved communities where no diagnostic infrastructure was previously available, enabling real-time monitoring of disease and surveillance trends in previously unmonitored regions. Trend analysis facilitated real-time monitoring of high-risk clusters with significantly changing disease and surveillance statuses. This study showcases a data-driven, evidence-based framework for neglected and emerging zoonotic disease surveillance in economically constrained regions.

**Supplementary Information:**

The online version contains supplementary material available at 10.1038/s41598-026-55346-7.

## Introduction

Rabies is transmitted mainly through the bite of an infected mammal and is almost always fatal once clinical symptoms appear. It accounts for an estimated 70,000 human deaths each year, with 99% of these deaths attributed to bites from domesticated dogs^1,2^. In high-income countries, mass dog vaccination has led to the elimination of dog-mediated rabies. However, 95% of rabies deaths occur in marginalized and impoverished communities in low- and middle-income countries (LMICs) across Asia and Africa^3^.

Successful elimination of dog-mediated rabies requires large-scale, coordinated public health and veterinary measures. These include high animal vaccination coverage (typically up to 70%), easy access to post-exposure prophylaxis (PEP) in humans, robust surveillance capacity, and active community and stakeholder engagement. Common barriers to rabies control and prevention include the absence of routine dog vaccination, limited access to rabies vaccines, and a shortage of veterinarians and trained personnel^4^. Even when resources are available, they may not be evenly distributed across high disease-burden areas within a country due to several limiting factors, such as socioeconomic disparities, political instability, public health infrastructure limitations, and geographic inaccessibility, which significantly contribute to the challenges faced in rabies control and prevention efforts within LMICs. This inconsistent distribution can lead to the reintroduction and reestablishment of rabies, mainly through porous geographic and political boundaries, even in regions with good rabies management policies^5^. It is, therefore, essential to implement an evidence-based surveillance system to monitor spatiotemporal trends in disease, quickly detect rabies outbreaks, and alert programs when surveillance efforts are inadequate in high-risk areas. Such systems not only aid in prioritizing high-risk communities based on resource availability but also help prevent resource wastage in regions where interventions are not needed.

Predictive models have been used to assess the risk of rabies in biting animals, where laboratory-based diagnosis is not always possible. Logistic regression (LR) is the commonly used classification technique to predict rabies probabilities in biting animals using clinical signs and case investigation outcomes^6,7^. However, LR and other conventional statistical techniques have stringent assumptions for data distributions which limit their applicability in many situations. Lately, machine learning-based classification techniques are gaining popularity for infectious disease prediction modeling because of their ability to handle large and complex epidemiological data to generate highly accurate predictions^8,41^. Machine learning (ML) is a branch within the field of artificial intelligence that focuses on developing algorithms and models capable of independently learning and improving at tasks through data and experience, without being directly programmed by humans^9^. ML-based ensemble models, such as extreme gradient boost (XGB), are widely used in healthcare and infectious disease research due to their ease of implementation and interpretation^10–13^. These techniques can be automatized with minimal human involvement to drastically decrease the human resources and time required to analyze surveillance data, which is key for real-time, operational decision-making.

In our previous study, we compared XGB with LR and demonstrated its utility for producing accurate and reliable predictions that can help support rabies risk assessment in animals^14^. In this study, we combine XGB risk modeling along with a graph pruning-based technique to identify spatiotemporal clusters, moving the analysis from individual animal risk to population-level transmission chains. This framework allows for the identification of active clusters in areas lacking diagnostic infrastructure, detecting outbreaks that would otherwise remain invisible while simultaneously monitoring surveillance efforts to flag anomalies in near-real time at the field level. Finally, we provide a comprehensive decision-support tool to differentiate between genuine disease trends and fluctuations in surveillance capacity, which could ultimately aid local program officers in real-time decision-making.

## Methodology

**Fig. 1:**
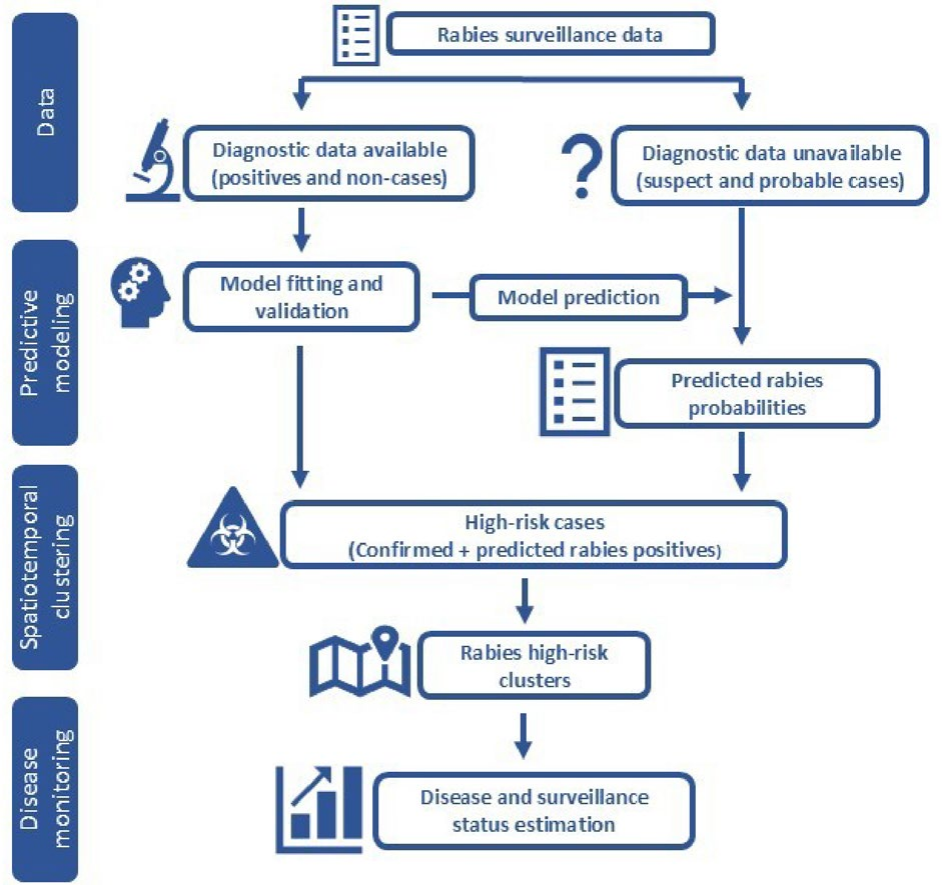
The flowchart for the various data preparation and analytical steps involved in the study.

The flowchart for the various data preparation and analytical steps involved in this study is presented in Fig. 1. All methods were carried out in accordance with relevant guidelines and regulations. All methods are reported in accordance with ARRIVE guidelines (https://arriveguidelines.org).

### Data

Haiti’s national rabies program follows an advanced form of surveillance called Integrated Bite Case Management (IBCM). The IBCM is a multi-sectoral approach to address rabies and animal bites which reduces human and animal rabies mortality through animal quarantine, bite-victim counseling, and vaccination tracking^15^. The electronic data related to potential rabies exposures are collected by field investigators using the Rabies Exposure Assessment and Contact Tracing (REACT) app^15^. The dataset consisted of information on event notification, geolocation, animal health investigation, rabies exposure investigation, animal quarantine, and diagnostic test results, if available.

For this study, we used REACT data collected between June 2018 and December 2023. Animal investigations with complete case histories and geographic locations were included in this analysis. All animals that had diagnostic confirmation of rabies virus either via Direct Fluorescent Antibody Test or Lateral Flow Test were categorized as confirmed positives^16^. The animals that tested negative or passed a 10-day observation period were classified as non-cases. The remaining cases that did not have a confirmed case status were grouped as unconfirmed cases. The analysis did not include any human rabies cases that might have occurred due to animal exposures.

### Predictive modeling

We used an XGB classifier to estimate the probability of rabies outcomes in the investigated animals using case history and clinical signs data. The XGB is an ensemble-based machine learning technique that gradually and sequentially produces hundreds of low-accuracy decision trees and combines them into a single highly accurate prediction model^17^. Data related to all animals with confirmed rabies status were used to train the rabies prediction model. The dataset was randomly split into training and validation sets at a ratio of 80:20. The input features used for prediction were derived from animal case history and clinical signs data collected during routine IBCM investigations. These included demographic markers (species and age), vaccination status, and pathognomonic clinical signs of rabies, such as aggression, hypersalivation, paralysis, vocal changes, and lethargy. Additionally, the model incorporated epidemiological risk factors, including whether the animal had bitten multiple people or animals and its clinical outcome (alive or dead) at the time of investigation.

Due to the relative rarity of rabies in biting animals (typically < 5%), our surveillance data had more non-cases compared to rabies cases. This imbalanced data could lead to a classifier model that fails to distinguish between positive and negative cases. Furthermore, the classifier could produce a deceptively high accuracy metric even if it wrongly predicts almost all instances as the negative class in case of a rare disease event^18^. To fix this imbalanced data issue in our data we used the Random oversampling (ROS) technique to rebalance our training data^19^. For model fitting, a grid search with 5-fold cross-validation was performed on the oversampled training data to obtain the optimal combination of hyperparameters. The grid search parameters included maximum depth (max_depth), minimum child weight (min_child_weight), number of estimators (n_estimators), learning rate (learning_rate), subsample ratio (subsample), the subsample ratio of columns (colsample_bytree), gamma (gamma), and L2 regularization term (reg_lambda). These hyperparameters were selected to prevent the model from learning noise and overfitting, optimizing tree complexity and regularization to ensure it remains robust to outliers and variations inherent in field-reported surveillance data. The model predicted rabies probability ranging between 0 and 1 as an outcome variable. Finally, we used isotonic regression to calibrate the obtained rabies probabilities^20^. The detailed description including the data-balancing, XGB model building, and probability calibration techniques used along with their predictive performance are described in detail elsewhere^14^. The contribution of predictor variables to model output was assessed using SHAP (SHapley Additive exPlanations) values^21^. Model evaluation was performed using Sensitivity (SN), Specificity (SP), and Accuracy (AC) Precision-Recall Area Under the Curve (PR-AUC), Positive Predictive Value (PPV) and Negative Predictive Value (NPV) for different predicted probability thresholds. To ensure the robustness of these estimates, 95% confidence intervals (CI) were calculated for all performance metrics using 1,000 bootstrap iterations on the independent validation set.

The best-fitted XGB model was used to predict the rabies probabilities of all unconfirmed cases. A risk stratification threshold of predicted probabilities was set up to categorize investigated cases into predicted positives or not. For our study, all unconfirmed cases with a predicted probability of more than the threshold were classified as predicted positives. The threshold value was selected based on model evaluation metrics to minimize the misclassification of confirmed rabies cases as lower risk along with subject matter opinion. Finally, we combined confirmed positives and predicted positives obtained via diagnostic confirmation and model prediction respectively, to obtain our rabies high-risk cases.

### Spatiotemporal cluster analysis

The spatiotemporal clusters were estimated using the graph pruning technique as described by Cori et al., 2019 ^22^. The spatiotemporal patterns of both confirmed positive cases and high-risk cases (i.e., confirmed positives + predicted positives) were analyzed using the clustering technique. This technique utilizes pairwise distance-based cutoffs to associate cases possibly connected to each other as part of a disease transmission chain. The pairwise distances were temporal distances defined by serial distance and spatial distances defined by spatial kernel. The cases are considered to be part of a cluster only if they are related both spatially and temporally to each other. The technique also accounts for underreporting of cases that could have gone unobserved in the disease transmission chain. For our analysis, we assumed a gamma-distributed rabies serial interval with a mean of 24 days (standard deviation 21 days)^23^, an average transmission distance of 0.88 km , and pruning cutoffs 98% quantile for the input distance distributions an assumed reporting rate of 2% for rabies-positive cases and 5% for model-derived high-risk cases.

### Trend analysis and status estimation

We used the Modified Mann–Kendall (MMK) test^24^ and Sen’s slope test^25^ to detect significant trends in the high-risk cases and percent high-risk cases for each identified cluster. The non-parametric MMK test statistically assesses monotonic upward or downward trends over time, where a positive Z score indicates an upward trend and a negative Z score indicates a decrease. Complementary to this, Sen’s slope calculates the magnitude and velocity of these temporal shifts. A 6-month evaluation window, spanning July to December 2023, was prioritized to simulate a real-time deployment scenario. This duration was selected to facilitate immediate situational awareness rather than a long-term retrospective study, using a significance level of 0.05 to evaluate shifts in both raw high-risk case counts and their percentages.

To separate biological changes in rabies transmission from fluctuations in reporting effort, we utilized the Situational Awareness for Infectious Diseases (SAID) algorithm (Table 1). This framework assesses disease and surveillance status by comparing trends in the absolute number of predicted high-risk cases with the percentage of those cases. By focusing on this proportion, we can detect outbreaks even when overall surveillance activity is declining. For instance, a stable case trend paired with an increasing percent case trend indicates a *disease increase* occurring alongside a *surveillance decrease*; this suggests that the disease is becoming more prevalent despite reduced reporting effort. Conversely, a stable case trend paired with a decreasing percent case trend signals a *disease decrease* occurring alongside a *surveillance increase*, indicating that we are finding the same number of cases only because we are investigating a larger volume of animals. This approach ensures that shifts in reporting capacity do not mask or mimic underlying biological trends.

**Table 1:** The Situational Awareness for Infectious Diseases (SAID) algorithm used to monitor disease and surveillance status based on absolute case and percent case trend of rabies. The case and percent case trend of the time series data are estimated using the Modified Mann–Kendall (MMK) test.

Data Input: Case trend // Percent case trend		Interpretation: Disease status		
		Increasing	Stable	Decreasing
**Interpretation: Surveillance status**	**Increasing**	Increase // Stable	Increase // Decrease	Stable // Decrease
	**Stable**	Increase // Increase	Stable // Stable	Decrease // Decrease
	**Decreasing**	Stable // Increase	Decrease // Increase	Decrease // Stable

## Results

Between June 2018 and December 2023, 13,403 rabies investigations were reported through the REACT application. Among them, 6,481 investigations had rabies status as either confirmed (96) or non-case (6,385) whereas the remaining 6,922 had an unconfirmed case status. A descriptive summary of animal characteristics and clinical presentation of investigated cases is presented in Table 2. The spatial and temporal distributions of rabies confirmed positives, non-cases, and unconfirmed cases are presented in Supplemental Figures S1 and S2, respectively.

### Model evaluation

The predictive performance of the XGB model was evaluated using an independent 20% validation (holdout) dataset for probability cut-off values ranging from 0.1 to 1. The evaluation metrics at 0.5 and 0.85 predicted probability cutoff are presented in Table 3. For our study, a probability threshold of 0.85 was used to classify cases as predicted positives, as it achieved near-perfect specificity (0.99, 95% CI: 0.98–0.99) and NPV (1.00, 95% CI: 0.99–1.00) while maintaining acceptable sensitivity (0.78, 95% CI: 0.61–0.95) for an early warning system in a resource limited settings (Table 3). The SHAP feature importance analysis identified aggression, animal health outcome, age, and vaccination status as the most influential features driving the model’s rabies risk predictions (Supplemental Figures S3).

**Table 2:** Descriptive summary of animal characteristics and clinical presentation of investigated cases.

Feature	Confirmed Positive (*N* = 96)	Non-case (*N* = 6385)	Unconfirmed (*N* = 6922)
**Age**			
Infant	2 (2.1%)	92 (1.4%)	41 (0.6%)
Junior	21 (21.9%)	2982 (46.7%)	1383 (20.0%)
Adult	65 (67.7%)	3176 (49.7%)	3390 (49.0%)
Unknown	8 (8.3%)	135 (2.1%)	2108 (30.5%)
**Vaccination status**			
Yes	6 (6.2%)	2194 (34.4%)	915 (13.2%)
No or not reported	90 (93.8%)	4191 (65.6%)	6007 (86.8%)
**Species**			
Dog	90 (93.8%)	6107 (95.6%)	6353 (91.8%)
Cat	2 (2.1%)	125 (2.0%)	181 (2.6%)
Others	4 (4.2%)	153 (2.4%)	387 (5.6%)
**Hypersalivation**			
Yes	33 (34.4%)	14 (0.2%)	65 (0.9%)
No or not reported	63 (65.6%)	6371 (99.8%)	6857 (99.1%)
**Paralyzed**			
Yes	11 (11.5%)	16 (0.3%)	38 (0.5%)
No or not reported	85 (88.5%)	6369 (99.7%)	6884 (99.5%)
**Lethargy**			
Yes	14 (14.6%)	20 (0.3%)	67 (1.0%)
No or not reported	82 (85.4%)	6365 (99.7%)	6855 (99.0%)
**Vocal change**			
Yes	23 (24.0%)	8 (0.1%)	34 (0.5%)
No or not reported	73 (76.0%)	6377 (99.9%)	6888 (99.5%)
**Aggression**			
Yes	72 (75.0%)	53 (0.8%)	1410 (20.4%)
No or not reported	24 (25.0%)	6332 (99.2%)	5512 (79.6%)
**Bite history**			
Bit ≥ 2 people/animals	9 (9.4%)	595 (9.3%)	585 (8.5%)
No or not reported	87 (90.6%)	5790 (90.7%)	6337 (91.5%)
**Animal health outcome**			
Dead during investigation	72 (75.0%)	242 (3.8%)	488 (7.0%)
No or not reported	24 (25.0%)	6143 (96.2%)	6434 (93.0%)

**Table 3:** The model evaluation metrics of the XGR-ROS model for a probability threshold of 0.5 and 0.85.

Evaluation metric	Cutoff value 0.5 (95% CI)	Cutoff value 0.85 (95% CI)
Accuracy	0.96 (0.95–0.97)	0.98 (0.98–0.99)
Specificity	0.96 (0.95–0.97)	0.99 (0.98–0.99)
Sensitivity	0.96 (0.87–1.00)	0.78 (0.61–0.95)
Precision-Recall Area Under the Curve	0.68 (0.49–0.86)	0.68 (0.47–0.86)
Negative Predictive Value	1.00 (1.00–1.00)	1.00 (0.99–1.00)
Positive Predictive Value	0.33 (0.21–0.44)	0.53 (0.37–0.71)

### Spatiotemporal cluster analysis

**Fig. 2:**
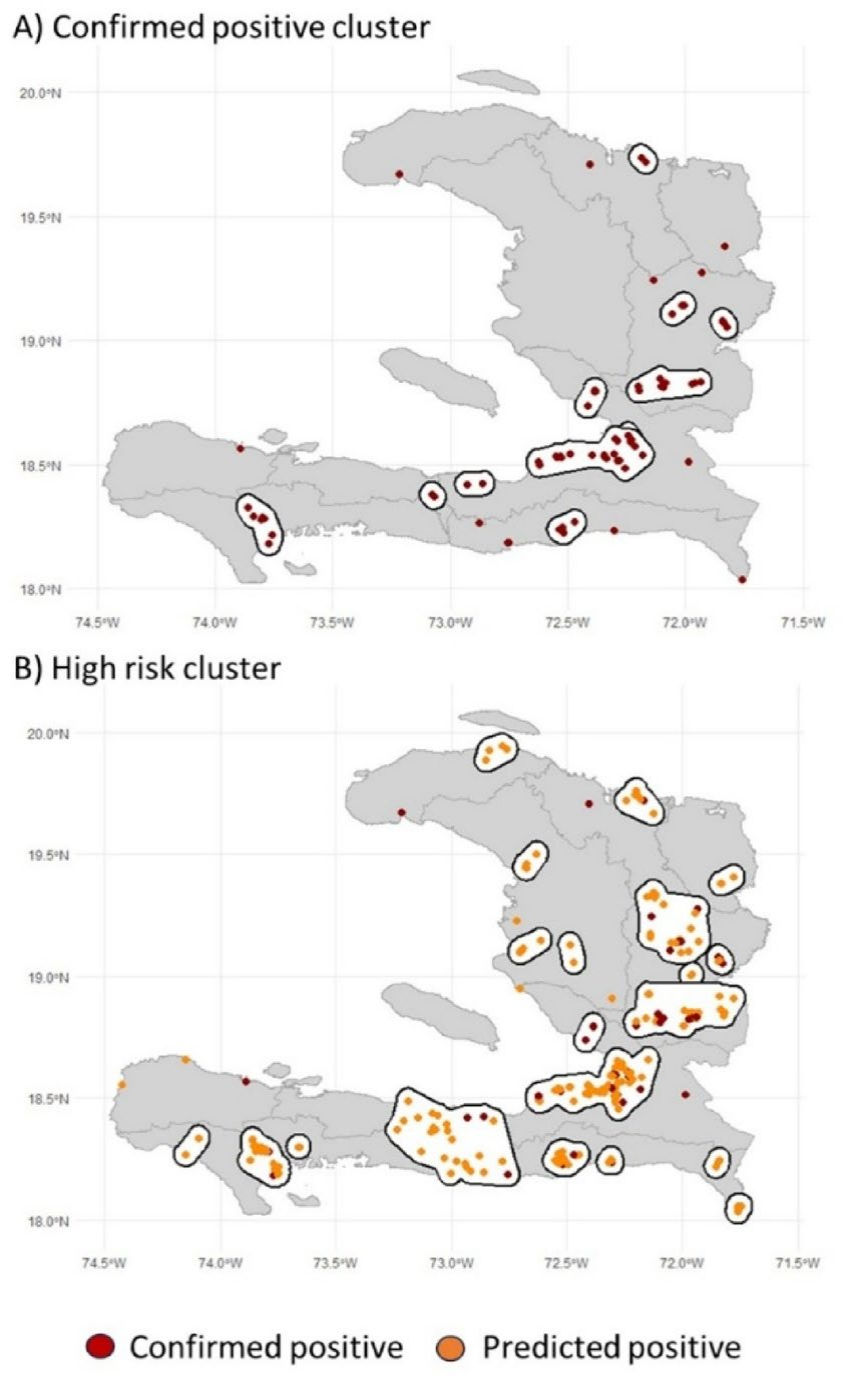
Maps of Haiti showing (**A**) laboratory confirmed positive clusters and (**B**) high-risk (laboratory confirmed + predicted positive) clusters. The maps were generated using R version 4.3.1 (www.r-project.org).

A total of 1,290 high-risk cases (96 confirmed cases, 1,194 model predicted cases) were used for rabies spatiotemporal cluster analysis in Haiti. The comparative maps depicting the clusters obtained using only the confirmed positive rabies cases and high-risk cases are shown in Fig. 2. Using the high-risk data, we identified 20 rabies clusters spread across Haiti, including 8 (40%) additional clusters that were not detected using confirmed positive data only. Supplemental Figure S4 shows the changes in shape and size of rabies high-risk clusters over three consecutive years (i.e., 2021, 2022, 2023). The largest cluster was located in the Ouest (cluster 3, *n* = 730), Sud (cluster 8, *n* = 149), and Center (cluster 6, *n* = 68) departments. The time series plots of these clusters showing high-risk and percent high-risk cases are presented in Supplemental Figure S5. Additionally, 9 singletons were identified that were not part of any cluster.

### Disease monitoring

**Fig. 3:**
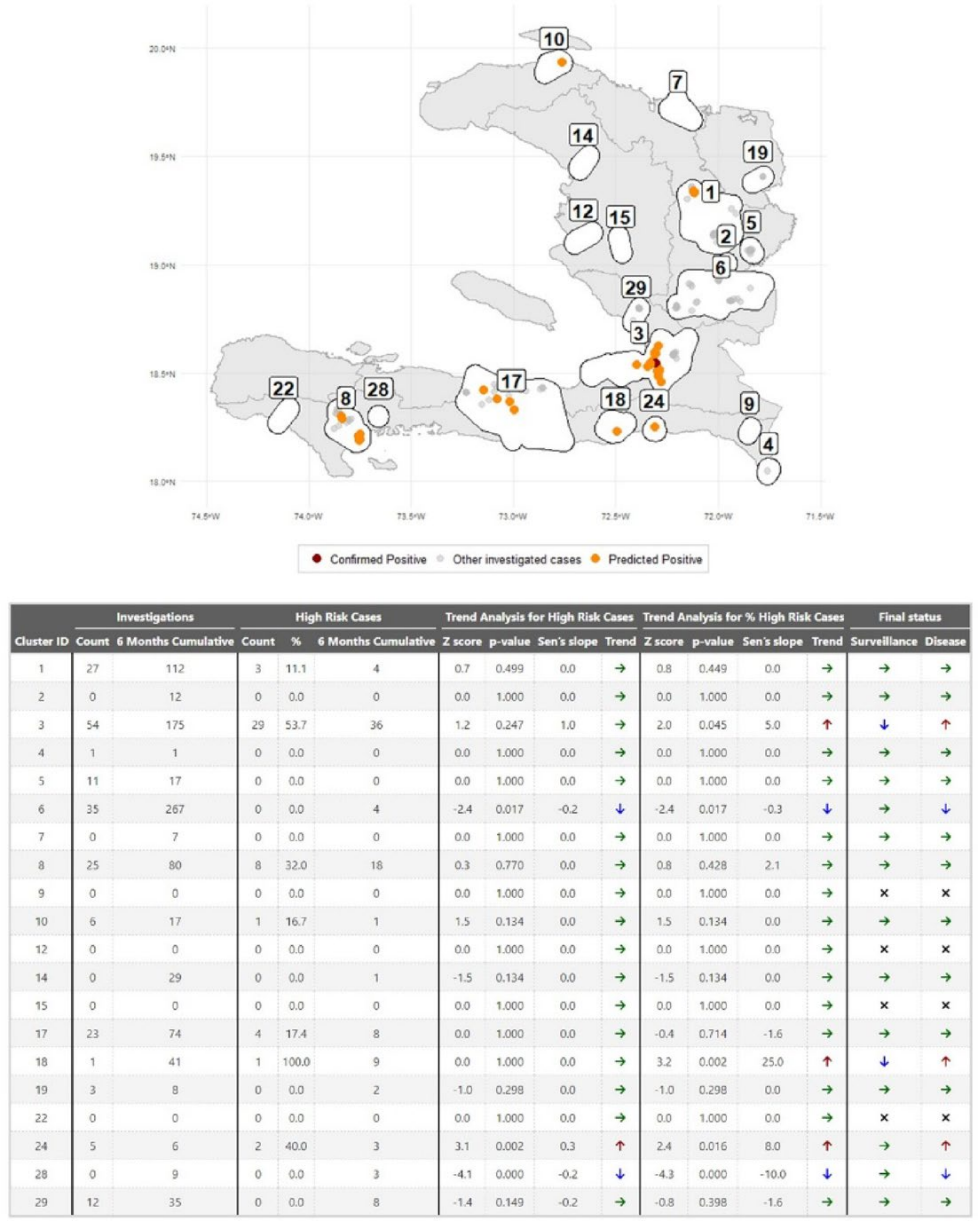
The rabies disease and surveillance status for December 2023 in Haiti. Top: Map of Haiti showing rabies high-risk clusters (orange dots), and investigations cases (gray dots) for December 2023. Bottom: The high-risk and percent high-risk case trends for December 2023 estimated using the MMK test and Sen’s slope test using 6-month historical data. The final rabies disease and surveillance status were calculated as per Table 2. The directionality of the arrows represents increasing (↑), decreasing (↓), and stable (→) case and percent high-risk trends along with final disease and surveillance status. The clusters with no investigations conducted in the past 6 months did not have a final status and were marked “**X**”. The maps were generated using R version 4.3.1 (www.r-project.org).

The high-risk and percent high-risk case trend as estimated using MMK and Sen’s slope tests and subsequently estimated surveillance and disease status using the SAID algorithm for each rabies cluster in Haiti for December 2023 is presented in Fig. 3. Cluster 3 and 18 showed an increasing disease status while the surveillance decreased in the past 6 months. On the other hand, Cluster 6 and 28 showed a decreasing disease status while the surveillance status remained stable. No investigations were conducted in clusters 9, 12, 15, and 22 in the last 6 months of the study period, resulting in an unknown status. The disease and surveillance status remained mostly stable in the remaining clusters.

## Discussion

Implementing effective rabies control and prevention programs requires accurate and reliable situational awareness and disease monitoring strategies. However, inadequate surveillance and diagnostic capabilities in LMICs hinder crucial public health decision-making. This often leads to the misallocation of limited resources, resulting in prioritization of lower-risk regions while overlooking high-risk areas. In this study, we utilized XGB, a machine learning technique, to classify high-risk rabies cases where the available diagnostic resources are scarce. We employed a graph-based spatiotemporal clustering method to identify high-risk rabies clusters. Finally, we applied nonparametric trend analysis and outbreak detection techniques to enhance the surveillance capability of rabies across large program areas.

The integration of predictive modeling and spatiotemporal clustering addresses several limitations inherent in traditional rabies surveillance methodologies. Traditional spatial analyses in rabies research frequently utilize heat maps or density-based approaches to visualize hotspots^26–28^. While effective for descriptive visualization and epidemiology, these methods are often static and do not distinguish between independent introductions and active, sustained transmission chains. Similarly, Ecological Niche Modeling (ENM) predicts potential disease persistence based on environmental suitability to understand the long-term biogeography of infectious diseases^29–31^. However, ENM often lacks the temporal sensitivity required for real-time outbreak detection because it emphasizes isolated components, such as ecological suitability or vector dynamics, without integrated, real-time temporal resolution. Furthermore, The implementation of public health programs is closely tied to governance structures and political boundaries^32^. However, infectious diseases like rabies often span multiple administrative jurisdictions, each with differing governance systems, program objectives, and resource availability. Strict and diligent rabies management efforts in one jurisdiction may fail to eliminate the disease if rabid animals are being introduced from neighboring areas^33,34^. These introductions could lead to emergence and large-scale outbreaks in previously rabies-free regions. Our spatiotemporal cluster analysis of confirmed positive and high-risk rabies cases reveals several large clusters in Haiti, spanning multiple communes and divisions. Identifying these clusters can support the establishment of centralized rabies management actions while allowing implementation to be delegated to the local level.

Identifying and monitoring the spatial and temporal dynamics of rabies cases is crucial for a successful rabies control and prevention strategy. Ideally, a surveillance program would identify the majority of rabid animals through investigation-based surveillance and gold-standard diagnostic tests. However, even the most well-funded and advanced programs likely detect only 1–5% of all animal rabies cases^35,36^. Our findings indicate that while the clustering methodology is sensitive to reporting rates, with cluster granularity increasing as reporting levels rise (Supplemental Figure S6), the hotspots identified at the 1–5% range remain a robust and authentic reflection of actual spatio-temporal dynamics.

In low- and middle-income countries (LMICs), barriers such as a high population of free-roaming dogs, a lack of trained professionals, limited diagnostic facilities, and weak transportation infrastructure reduce the number of investigations with confirmatory diagnoses. As a result, relying solely on confirmed rabies cases often underestimates the true disease burden. By incorporating model predictions into our spatiotemporal cluster analysis, we identified eight additional pockets of potential rabies transmission that would have gone undetected if only confirmed positive cases were used. Many of these clusters were located in communes that did not consistently perform rabies testing, despite routinely investigating animal bite cases. Additionally, we identified singleton cases, isolated instances not yet part of a transmission chain, that could potentially spark larger outbreaks. Our technique can aid in tracking the spatiotemporal evolution of rabies epidemiology, rapidly identifying high-risk areas, and directing limited resources such as diagnostic kits, vaccines, and trained field investigators to contain or mitigate local outbreaks.

In our study, we selected a high rabies probability threshold of 0.85 to classify cases as high-risk. This choice resulted in very high specificity (0.99, 95% CI: 0.98–0.99) and negative predictive value (1.00, 95% CI: 0.99–1.00), demonstrating strong confidence in the model’s ability to rule out negative cases. These metrics are particularly valuable in rabies surveillance programs within endemic regions, where dog bites are frequent, yet less than 1–5% of bites are estimated to involve rabid dogs^37,38^. However, higher probability thresholds come at the cost of reduced SN (0.78, 95% CI: 0.61–0.95), meaning some true positive cases may be misclassified as negatives. For decisions related to post-bite patient treatment, this sensitivity would not be adequate to ensure appropriate care. A lower cutoff of around 0.5 may be more appropriate if a model such as this were used to inform patient management decisions. This lower cutoff improves SN to 0.96 (95% CI: 0.87–1.00), equivalent to many validation studies for gold standard rabies diagnostics (Table 3, Supplemental Table S1). For surveillance and response decision-making, a higher threshold for case determination (e.g. 0.85) can ensure that limited resources for rabies response are allocated where they are most needed. A cluster with a high number of predicted positive cases could serve as an indicator of a potential rabies outbreak, warranting additional monitoring and allocation of necessary resources to verify the genuine public health risk, ensure diagnostic confirmation, and prepare for response. This system can facilitate a rapid response in the form of dog vaccinations and public awareness in case of confirmed cases in a cluster.

In rabies-endemic settings like Haiti, the prevalence of laboratory-confirmed rabies is typically very low, representing a small fraction of total animal investigations^39^. To resolve this, we employed a data-balancing strategy that requires the model to more frequently encounter rabies outcomes during its learning phase. By doing so, we successfully increased the model’s ability to correctly identify high-risk cases that might otherwise be overlooked. Although such balancing techniques can occasionally skew the posterior probabilities of a classifier, we corrected for this using probability calibration. This balanced approach allows the framework to achieve high sensitivity without sacrificing the reliability of the risk scores used to trigger local public health responses.

Multiple factors, including the availability of financial and technical resources, influence the consistency and stability of a rabies surveillance program. Therefore, it is crucial to monitor fluctuations in rabies cases within a given programmatic area in relation to the total number of investigations conducted. For instance, a rabies cluster might experience a sharp increase in cases if there is a sudden surge in reported investigations. Conversely, the same cluster could show fewer cases if there is a drop in surveillance capacity. In our study, we analyzed monthly trends in high-risk cases and the percentage of high-risk cases within each cluster, assessing their statistical significance using the MMK test. Sen’s slope provided the magnitude of these trends. Finally, the SAID algorithm offered a comprehensive approach to monitoring disease and surveillance status, prioritizing high-risk areas while accounting for inherent bias introduced by fluctuating surveillance efforts, and updating control and prevention strategies in a timely manner. As shown in our analysis (Fig. 3) Clusters 1 and 2 demonstrated stable trends across both absolute high-risk counts and proportional risk, resulting in a *stable status* classification, indicating baseline surveillance and disease levels. In contrast, Clusters 3 and 18 were flagged with a *surveillance decrease* paired with a *disease increase*. In these clusters, the absolute number of high-risk cases remained stable, but the proportion of high-risk investigations significantly increased (*p* < 0.05). This suggests that while fewer animals were being investigated overall, a higher percentage of those encountered were likely rabid, indicating that declining surveillance effort may be masking an active biological outbreak.

While machine learning models are highly effective at learning complex patterns from the surveillance features, their overall predictive performance is fundamentally dependent on the quality of the input data. Our SHAP analysis identified aggression, animal health outcome, age, and vaccination status as the most influential features driving rabies risk predictions. Although biologically sound, the utility of these indicators is inherently limited by the consistency of field observations. Clinical signs like aggression are frequently subjectively interpreted based on an investigator’s experience, while vaccination status is largely unavailable for stray dogs. In cases involving owned animals, obtaining accurate vaccination status is hindered by the lack of formal pet registries and a reliance on owner recall, which could introduce recall bias into the dataset. Any spatial and temporal variations in data collection driven by differences in such investigator training, experience, regional accessibility, or local reporting practices can introduce noise and potential bias into the analytical outputs. To mitigate these risks and ensure the long-term reliability of this situational awareness framework, it is essential to institutionalize standardized training for all field officers and implement periodic data quality audits. Such measures are critical to minimizing collection bias and ensuring that the model’s predictions accurately reflect the underlying biological dynamics of rabies across all geographic regions.

The global rabies community has set an ambitious goal to eliminate human deaths from dog-mediated rabies by 2030 ^40^. However, tens of thousands of people continue to die from rabies each year, primarily in LMICs. Unfortunately, significant improvements in surveillance and diagnostic infrastructure are unlikely to occur in the near future. This creates an urgent need for rabies-endemic countries to adopt alternative techniques while healthcare and public health systems scale up. Our data-driven rabies monitoring and situational awareness framework can support the implementation of advanced surveillance systems by prioritizing high-risk areas and tailoring control and prevention strategies to address local conditions and challenges.

## Supplementary Information

Below is the link to the electronic supplementary material.

Supplementary Material 1

## Data Availability

The datasets used during the current study are available from the corresponding author on reasonable request.
