# Supplementary material for: Biosurveillance and early outbreak detection of rabies in settings with limited laboratory capacity using spatiotemporal clustering and a machine learning framework
Source: Sci Rep. 2026 May 28;16:24391. doi: 10.1038/s41598-026-55346-7 (PMC13448829; doi:10.1038/s41598-026-55346-7)
Supplement: Supplementary file 1 [file 41598_2026_55346_MOESM1_ESM.pdf]

Biosurveillance and Early Outbreak Detection of Rabies in Settings with Limited Laboratory Capacity Using Spatiotemporal Clustering and a Machine Learning Framework

Ravikiran Keshavamurthy[1]*, Jesse Blanton[1], Lillian Orciari[1], Andrew D. Gibson[3], Pierre Dilius[2], Haim Joseph[2], Ryan M. Wallace[1]

[1]Poxvirus and Rabies Branch, Division of High Consequence Pathogens and Pathology, National Center for Emerging and Zoonotic Infectious Diseases, Centers for Disease Control and Prevention, Atlanta, GA, United States

[2]Haiti Ministry of Agriculture, Rural Development and Natural Resources, Port au Prince, Haiti.

[3]Mission Rabies, Cranborne, Dorset, United Kingdom.

*Author to whom correspondence should be addressed.

* Ravikiran Keshavamurthy, Centers for Disease Control and Prevention, 1600 Clifton Rd NE, Atlanta, GA 30329-4027, USA

Email: mfl3@cdc.gov

Supplemental Material

Supplemental Table S1: The predictive performance of the XGB-ROS model was evaluated for probability cut-off values ranging from 0.1 to 1

Probability threshold	True positive	True negative	False positive	False negative	Sensitivity	Specificity	Accuracy	F1 score	PR-AUC	Positive predictive value	Negative predictive value
0.01	23	0	1274	0	1	0	0.02	0.03	0.69	0.02	-
0.02	23	0	1274	0	1	0	0.02	0.03	0.69	0.02	-
0.03	23	0	1274	0	1	0	0.02	0.03	0.69	0.02	-
0.04	23	0	1274	0	1	0	0.02	0.03	0.69	0.02	-
0.05	23	2	1272	0	1	0	0.02	0.03	0.69	0.02	1
0.06	23	2	1272	0	1	0	0.02	0.03	0.69	0.02	1
0.07	23	23	1251	0	1	0.02	0.04	0.04	0.69	0.02	1
0.08	23	251	1023	0	1	0.2	0.21	0.04	0.69	0.02	1
0.09	23	286	988	0	1	0.22	0.24	0.04	0.69	0.02	1
0.1	23	643	631	0	1	0.5	0.51	0.07	0.69	0.04	1
0.2	23	1215	59	0	1	0.95	0.95	0.44	0.69	0.28	1
0.3	23	1215	59	0	1	0.95	0.95	0.44	0.69	0.28	1
0.4	22	1243	31	1	0.96	0.98	0.98	0.58	0.69	0.42	1
0.5	22	1243	31	1	0.96	0.98	0.98	0.58	0.69	0.42	1
0.6	22	1243	31	1	0.96	0.98	0.98	0.58	0.69	0.42	1
0.7	19	1257	17	4	0.83	0.99	0.98	0.64	0.69	0.53	1
0.75	19	1257	17	4	0.83	0.99	0.98	0.64	0.69	0.53	1
0.85	19	1258	16	4	0.83	0.99	0.98	0.66	0.69	0.54	1
0.9	19	1261	13	4	0.83	0.99	0.99	0.69	0.69	0.59	1
1	0	1274	0	23	0	1	0.98	0	0.69	-	0.98

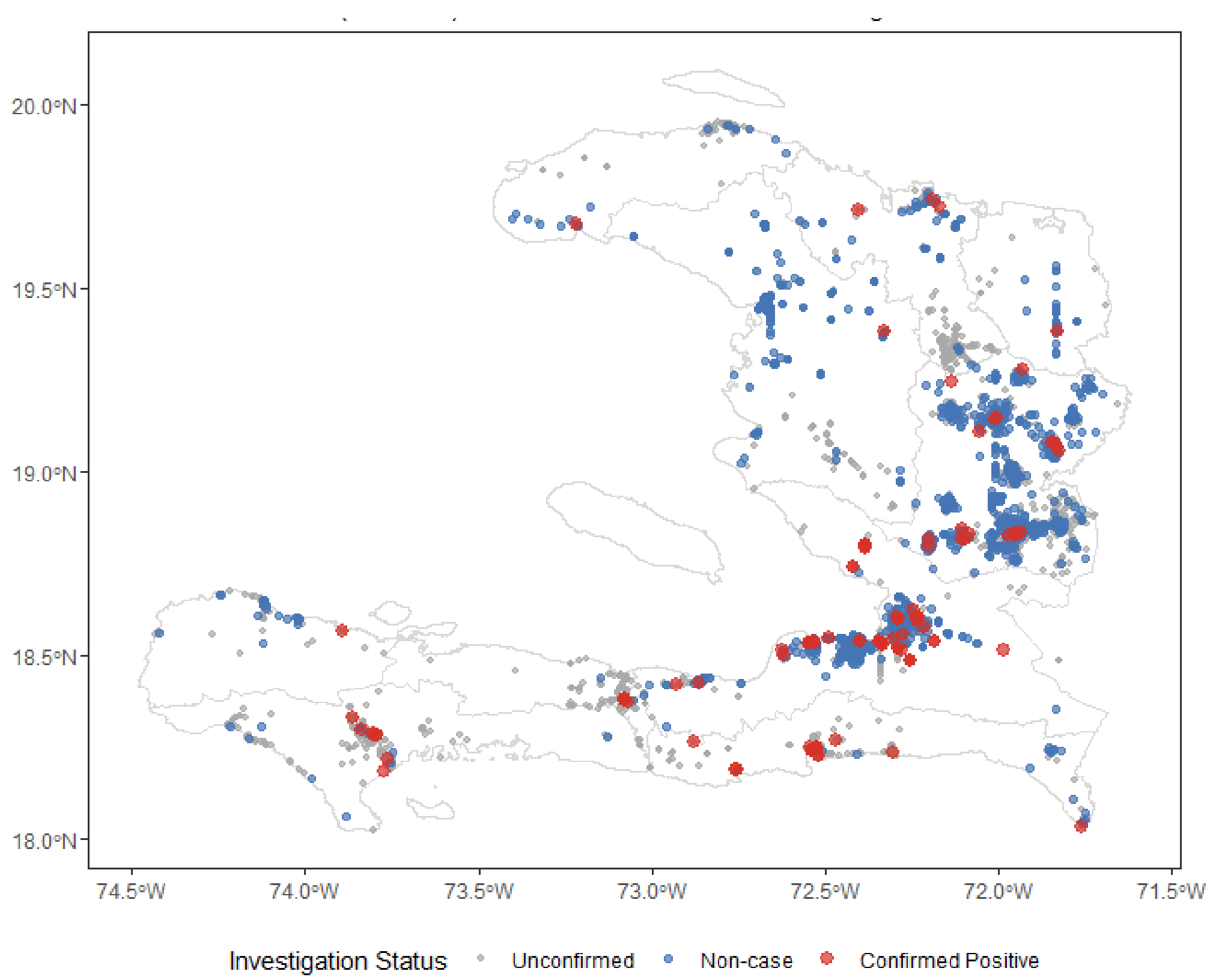

Supplemental Figure S1: Spatial distribution of rabies confirmed positives, non-cases, and unconfirmed cases between June 2018 and December 2023

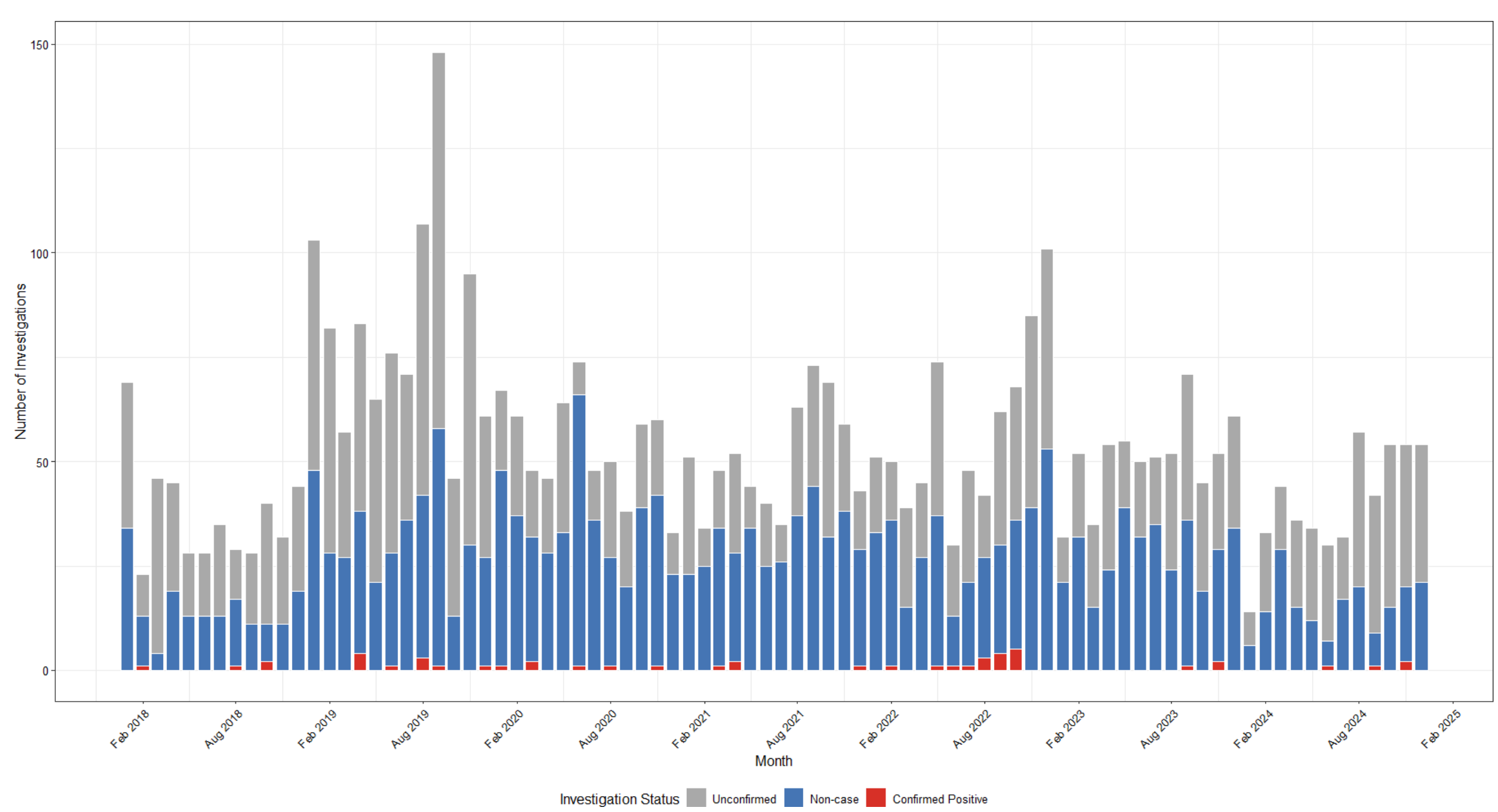

Supplemental Figure S2: Monthly temporal distribution of rabies confirmed positives, non-cases, and unconfirmed cases between June 2018 and December 2023.

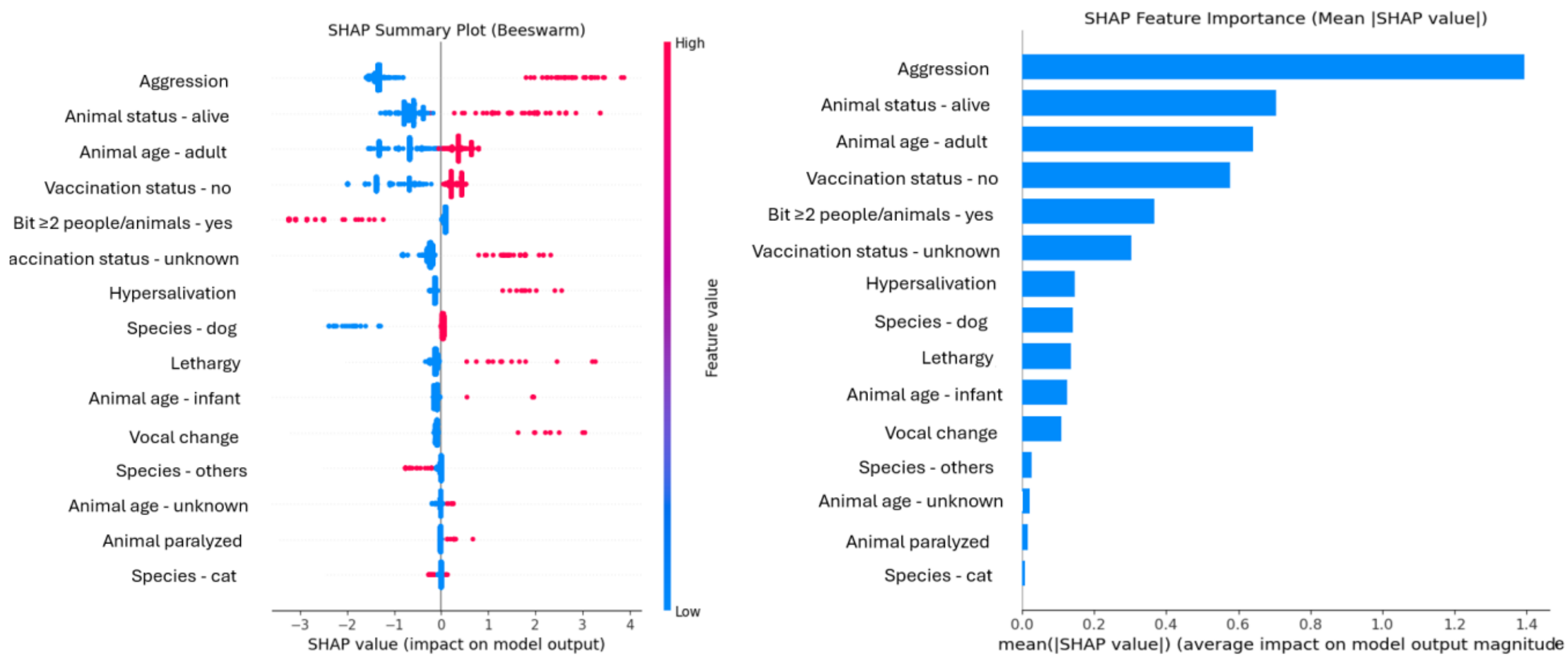

Supplemental Figure S3: Contribution of predictor variables to model output assessed by SHAP (SHapley Additive exPlanations) values. A) Beeswarm plot showing the distribution and direction of SHAP values for each feature across test-set observations (B) Bar chart showing mean absolute SHAP value per feature, representing overall contribution to model predictions.

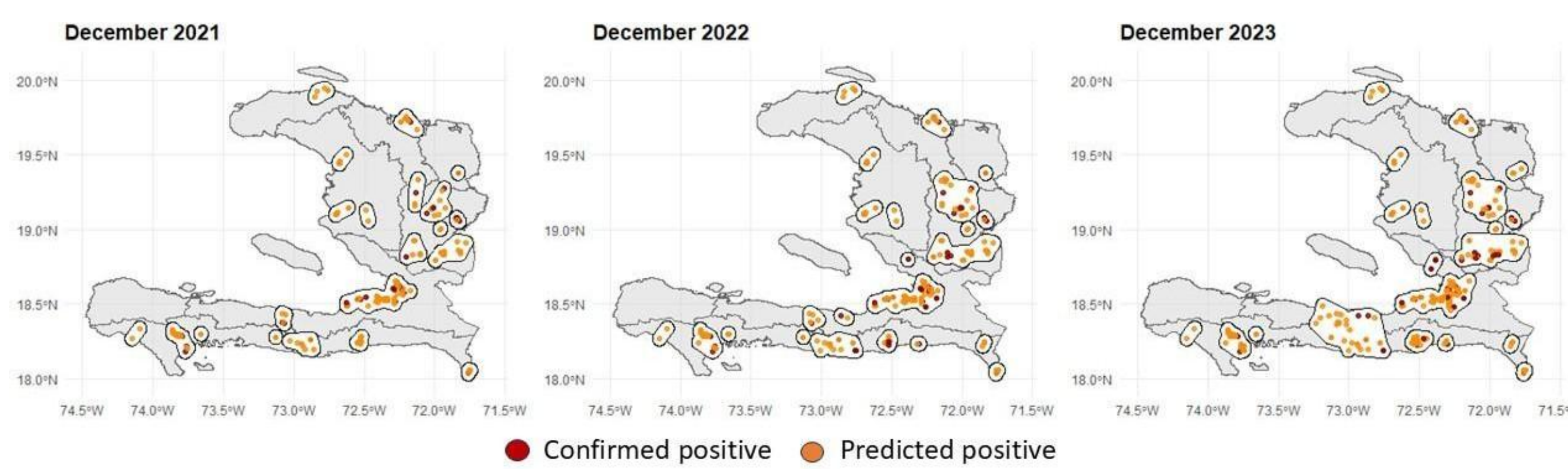

Supplemental Figure S4: Maps of Haiti showing changes in shape and size of rabies high-risk clusters in the three consecutive years (i.e., 2021, 2022, and 2023).

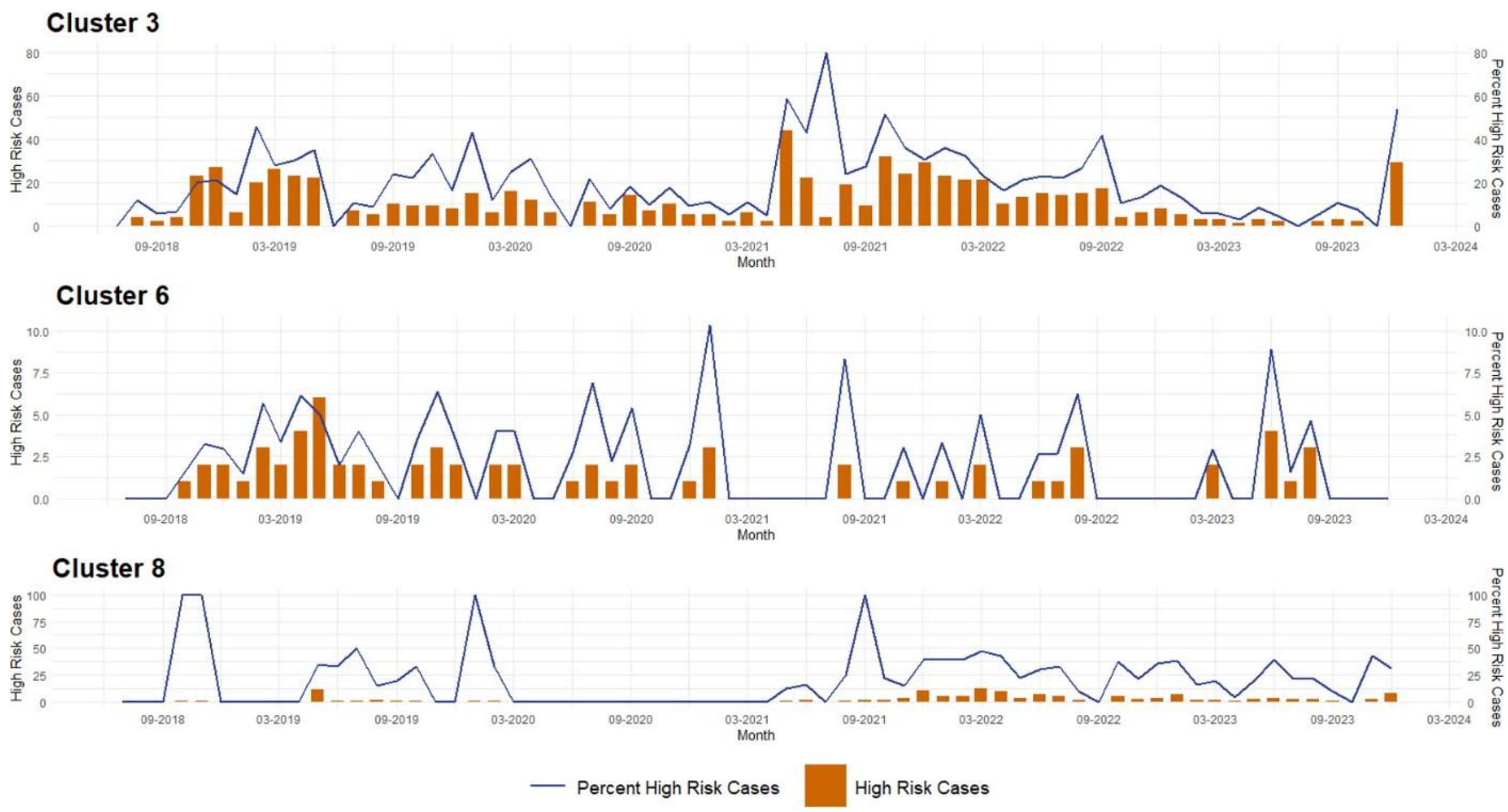

Supplemental Figure S5: The time series plots of high-risk and percent high-risk cases belonging to the three biggest clusters in three Haiti. The clusters were located in Ouest (cluster 3), Sud (cluster 8), and Center (cluster 6) departments.

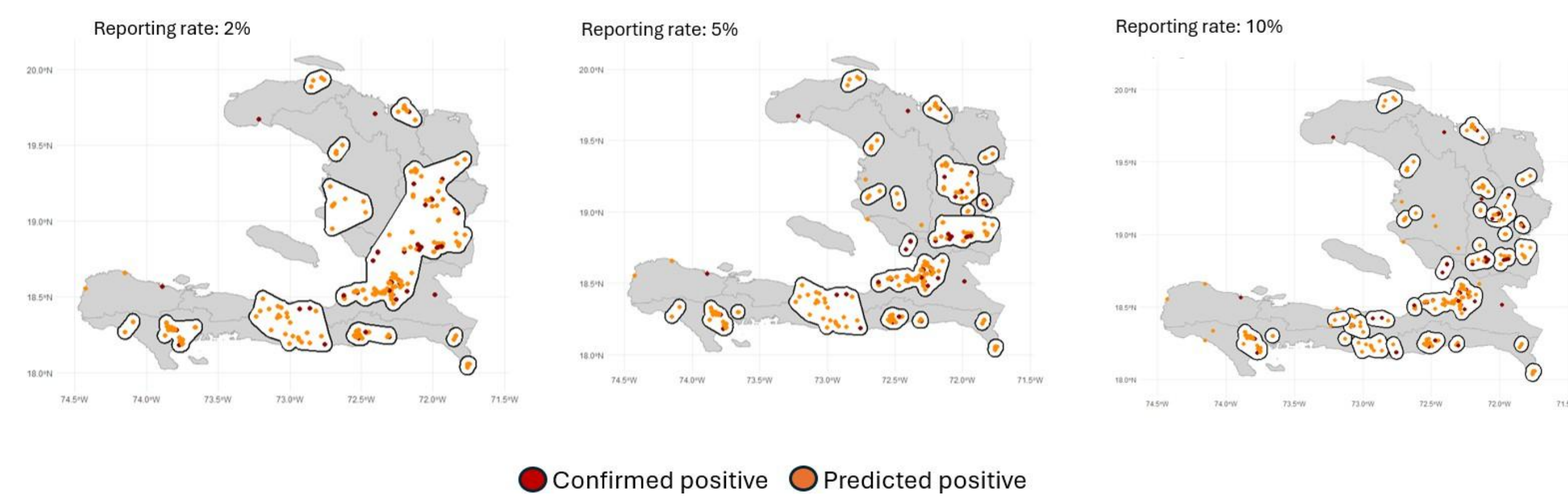

Supplemental Figure S6: Sensitivity analysis demonstrating high-risk rabies case distributions and derived clusters across overall reporting rates of 2%, 5%, and 10% in Haiti.
